# Dichotomous Keys Based on Cytogenetic Data for Triatomines Reported in Brazilian Regions with Outbreaks of Orally Transmitted Chagas Disease (Pernambuco and Rio Grande Do Norte)

**DOI:** 10.3390/tropicalmed8040196

**Published:** 2023-03-29

**Authors:** Denis Vinícius de Mello, Emercio Felisberto Nhapulo, Laura Poloto Cesaretto, Julia Junqueira Alevi, Daniel Cesaretto Cristal, Giulia Montanari, Cleber Galvão, Kaio Cesar Chaboli Alevi

**Affiliations:** 1 Instituto de Biociências, Universidade Estadual Paulista “Júlio de Mesquita Filho” (UNESP), Rua Dr. Antônio Celso Wagner Zanin, 250, Distrito de Rubião Júnior, Botucatu 18618-689, Brazil; 2 Laboratório de Entomologia em Saúde Pública, Departamento de Epidemiologia, Faculdade de Saúde Pública, Universidade de São Paulo, Av. Dr. Arnaldo 715, São Paulo 01246-904, Brazil; 3 Laboratório Nacional e Internacional de Referência em Taxonomia de Triatomíneos, Instituto Oswaldo Cruz (FIOCRUZ), Av. Brasil 4365, Pavilhão Rocha Lima, sala 505, Rio de Janeiro 21040-360, Brazil

**Keywords:** triatominae, taxonomy, cytokey

## Abstract

Chagas disease (CD) affects about eight million people worldwide. Brazil has the highest number of estimated cases and the largest number of deaths due to CD. Considering the recent outbreaks of oral CD involving at least 27 cases of acute CD in Pernambuco (PE) as well as 18 cases and 2 deaths in the Rio Grande do Norte (RN), we developed dichotomous keys for the identification of triatomine species in these Brazilian states based on cytogenetic data. All triatomine species could be distinguished by cytogenetic characteristics, emphasizing the importance of the newly developed taxonomic keys for the correct identification of triatomes from PE and RN, particularly for species that exhibit morphological similarities, such as *Triatoma brasilensis* and *T. petrocchiae* (present in both states) and *T. maculata* and *T. pseudomaculata* (as *T. pseudomaculata* has been misidentified as *T. maculata* in PE and RN). These alternative keys are expected to provide a useful tool for the scientific community and, above all, health agents, aimed at preventing mistakes from occurring in the identification of the vectors present in PE and RN related to CD outbreaks caused by oral infection.

## 1. Introduction

Chagas disease (CD) (also referred to as American trypanosomiasis) is a neglected disease caused by the protozoan *Trypanosoma cruzi* (Chagas, 1909) (Kinetoplastida, Trypanosomatidae) that affects about eight million people worldwide (although an estimated 70 million people are at risk of infection), resulting in approximately 10,000 deaths per year [1,2,3]. This disease is found mainly in endemic areas of 21 continental Latin American countries (where it kills more people each year than any other parasite-borne disease, including malaria [3,4]), although increasing cases have been reported in Canada and the United States of America, as well as in many European and some African, Eastern Mediterranean, and Western Pacific countries [1,2,3].

Brazil has the highest number of estimated cases and the largest number of deaths due to CD (7903 in 1990 and 6523 in 2019) [5]. CD can be transmitted by congenital transmission (transplacental), blood transfusions, organ transplantation, accidental laboratory exposure by triatomine vectors (Hemiptera: Triatominae), orally through breastfeeding, by the consumption of raw or undercooked meat from wild animals contaminated with *T. cruzi*, and by consumption of food that is contaminated with feces/urine from triatomines infected with the protozoan (such as juices and fruit pulp consumed in natura) [2]; the latter form of transmission is currently recognized as the main source of infection (especially in the Brazilian Amazon) [6,7]. However, it is worth mentioning that cases of oral transmission do not rule out the importance of triatomines, as food is often is contaminated with *T. cruzi* from the intestines of insects [1].

The Triatominae subfamily comprises 157 species; all 154 living species are considered to be potential CD vectors [8,9,10]. More than 60 species are present in Brazil, including species belonging to the genera *Alberprosenia* Martínez & Carcavallo, 1977; *Belminus* Stål, 1859; *Microtriatoma* Prosen & Martínez, 1952; *Parabelminus* Lent, 1943; *Cavernicola* Barber, 1937; *Psammolestes* Bergroth, 1911; *Rhodnius* Stål, 1859; *Eratyrus* Stål, 1859; *Panstrongylus* Bergroth, 1879; and *Triatoma* Laporte, 1832, more than half of which are endemic [11]. The northeast region includes about 30 species belonging to the genera *Cavernicola* Barber, 1937; *Eratyrus*, *Panstrongylus*, *Parabelminus*, *Psammolestes*, *Rhodnius,* and *Triatoma* [12].

In the states of Pernambuco (PE) and Rio Grande do Norte (RN), where outbreaks of oral CD have occurred [13,14], 13 species [*Panstrongylus geniculatus* (Latreille, 1811); *P. lutzi* Neiva & Pinto, 1926; *P. megistus* Burmeister, 1835; *P. tibiamaculatus* (Pinto, 1926); *Psammolestes tertius* Lent & Jurberg, 1965; *Rhodnius nasutus* Stal, 1859; *R. neglectus* Lent, 1954; *Triatoma brasiliensis* Neiva, 1911; *T. melanocephala* Neiva & Pinto, 1923; *T. petrocchiae* Pinto & Barreto, 1925; *T. pseudomaculata* Corrêa & Spínola, 1964; *T. rubrofasciata* (De Geer, 1773) and *T. sordida* (Stål, 1859)] and 9 species [*P. lutzi*, *P. megistus*, *P. tertius*, *R. nasutus*, *T. brasiliensis*, *T. melanocephala*, *T. petrocchiae*, *T. pseudomaculata* and *T. rubrofasciata*] have been reported to date, respectively [15,16,17].

The correct identification of these insects contributes directly to vector control programs, allowing the prioritization of species of primary importance during epidemiological surveillance [18]. Until 2019, classification was mainly based on dichotomous keys based on morphological data [15,19]. However, in some cases, phenotypic characters are not informative (such as phenotypic plasticity and cryptic speciation [20]), and alternative dichotomous keys using cytogenetic data (CytoKey) have been proposed [21,22,23,24].

In Brazil, several outbreaks of acute CD have been reported in which groups of individuals gathered in the same place, ingested the same food, and became sick almost simultaneously, with fever and general manifestations of a systemic infection [25]. These outbreaks occurred because triatomines infected with *T. cruzi* or the feces of these infected vectors were processed together with food consumed in natura [26,27]. At the end of the 20th century, 50% of acute CD cases recorded in the Amazon region, for example, were attributed to oral transmission [28]; at the beginning of the 21st century, this rate reached 70% [27].

In the Brazilian Northeast, research on CD outbreaks is not prioritized compared with other areas of Brazil, even though outbreak events are serious [7]. Outbreaks caused by oral contamination have already been registered in Paraíba (by sugarcane juice ingestion) [26], Bahia (possibly associated with fatal cases) [29], Ceará (possibly due to the consumption of contaminated vegetable soup) [30], Pernambuco (PE) (due to the consumption of contaminated food or beverages at a religious event) [14], and RN (where 2 deaths occurred after the consumption of contaminated food) [13].

Considering the recent outbreaks caused by oral infection resulting in at least 27 cases of acute CD in PE [14] and 18 cases and 2 deaths in the RN [13], we developed dichotomous keys to assist in the identification of triatomine species from the states of PE and RN based on cytogenetic data.

## 2. Materials and Methods

Cytogenetic data reported in the literature—including the chromosome number (as determined by lacto-acetic orcein staining), constitutive heterochromatin pattern (determined by C-banding), and 45S rDNA localization (probe analyzed with fluorescent in situ hybridization-FISH)—for triatomines present in PE and RN were revisited [31,32,33,34]. An identification key was developed based on Borsatto et al. [21,22].

## 3. Results and Discussion

Based on the cytogenetic characteristics of triatomines, we present dichotomous keys (CytoKeys) for the states of PE (Table 1) and RN (Table 2). All triatomine species could be distinguished by cytogenetic characteristics (Table 1 and Table 2).

Species of the genus *Panstrongylus* could be easily differentiated by the number of chromosomes (Table 1 and Table 2). *Panstrongylus geniculatus* and *P. tibiamaculatus,* which have the same number of chromosomes, could be distinguished by the constitutive heterochromatin pattern (Table 1). Species in the tribe Rhodniini could be distinguished by the heterochromatic pattern and by the composition of the chromocenter in prophase cells (Table 1 and Table 2). Finally, to differentiate among *Triatoma* spp., several cytogenetic characters were needed: the number of chromosomes, 45S rDNA localization, and properties of constitutive heterochromatin in chromosomes and prophase cells (Table 1 and Table 2).

*Triatoma brasiliensis* Neiva, 1911 and *T. petrocchiae* (Pinto & Barreto, 1925) belong to the *T. brasiliensis* subcomplex [35,36] reported in both states (Table 1 and Table 2). *Triatoma brasiliensis* is the most important vector of CD in semi-arid Brazil (frequently colonizing domiciles), and *T. petrocchiae* has not been associated with major outbreaks to date, as it is rarely found in habitats related to the peridomicile or intradomicile [37]. These species live in sympatry on rocky outcrops (where infection with *T. cruzi* has been reported) [37]. They exhibit morphological similarities [19,38] and can be distinguished by the following characters: i. presence of light stains on the femurs of *T. petrocchiae* [38]; ii. absence of a spongy pit in the tibiae of *T. petrocchiae* males; iii. presence of a practically glabrous rostrum and shorter first antennal segment in *T. petrocchiae;* and iv. membrane of hemelytra with a dark cloudy spot across the vein separating the two cells in *T. petrocchiae* [19]. These subtle differences can result in taxonomic errors in entomoepidemiological surveys, emphasizing the importance of the taxonomic keys for correct species identification.

Cytogenetic studies have provided insight into reproductive [39,40,41], physiological [42,43], evolutionary [44,45,46], systematic [34,47], and taxonomic [33,36,48] aspects of Triatominae. Cytotaxonomy was initially developed in 1950, when Schreiber and Pellegrino [49] described some karyotypes of species from South America. Since then, Ueshima [50] characterized new karyotypes and, in addition to chromosome number, used meiosis and the heteropyknotic pattern to differentiate 20 species of triatomines. More recently, the constitutive heterochromatin pattern [32,51,52] and the distribution of 45S rDNA probes [33,34,47] have been used to differentiate CD vectors.

The first identification keys for triatomines based on cytogenetic data were proposed by Borsatto et al. [21,22], who developed keys for species from the states of Alagoas, Amapá, Ceará, Roraima, Santa Catarina, and São Paulo (differentiating species from the genera *Panstrongylus*, *Psammolestes*, *Rhodnius*, and *Triatoma*). Recently, Oliveira et al. [24] reported, for the first time, *T. infestans* (Klug, 1834) in the state of Espírito Santo and developed a key based on cytogenetic data for all species reported in the state (differentiating species from genera *Panstrongylus*, *Cavernicola*, *Rhodnius* and *Triatoma*). Furthermore, Gonzalez-Britz et al. [23] performed an entomoepidemiological study of *T. sordida* (Stål, 1859) in Paraguay and presented a key based on cytogenetic data for all triatomines present in this Latin American country (differentiating species from the genera *Panstrongylus*, *Psammolestes* and *Triatoma*).

*Triatoma brasiliensis* and *T. pseudomaculata* Corrêa & Espínola, 1964 are the most abundant species in the states of PE and RN, where they are naturally infected by *T. cruzi* in anthropic areas [53,54]. Due to the morphological similarities between *T. pseudomaculata* and *T. maculata* (Erichson, 1848), until 1964 these taxa were considered the same species [55]. Lucena et al. [56] studied triatomines in Northeastern Brazil and reported the presence of *T. maculata* in PE and RN. However, the distribution of *T. maculata* in Brazil is restricted to Roraima [12,15], that is, its report in both states represents an error in the identification of *T. pseudomaculata* specimens. Although *T. maculata* is not included in the key (since it does not contribute to vector diversity in the RN), it can be easily differentiated by cytogenetic characteristics (*T. maculata*: 45S rDNA probe located in XY sex chromosomes; *T. pseudomaculata*: 45S rDNA probe located in one autosomal pair [47]).

## 4. Conclusions

We developed two dichotomous keys to assist in the correct identification of triatomines present in PE and RN, based on cytogenetic data. These alternative keys have practical implications for the scientific community and health agents, providing a basis for the prevention of errors in the identification of key vectors contributing to CD outbreaks caused by oral infection in these states.

## Figures and Tables

**Table 1:** Dichotomous key for species from Pernambuco, based on cytogenetic data.

Identification Key (CytoKey)	
1. Karyotype with 2n = 21 chromosomes (18A + X_1_X_2_Y)	*Panstrongylus megistus*
2. Karyotype with 2n = 23 chromosomes (20A + X_1_X_2_ Y)	3
3a. Prophase without heterochromatin blocks dispersed inside the nucleus	*Panstrongylus geniculatus*
3b. Prophase with heterochromatic blocks dispersed inside the nucleus	*Panstrongylus tibiamaculatus*
4. Karyotype with 2n = 25 chromosomes (22A + X_1_X_2_Y)	*Triatoma rubrofasciata*
5. Karyotype with 2n = 24 chromosomes (20A + X_1_X_2_ X_3_Y)	6
6a. 45S rDNA probe located in one autosomal pair	*Panstrongylus lutzi*
6b. 45S rDNA probe located in one X sex chromosome	*Triatoma melanocephala*
7. Karyotype with 2n = 22 chromosomes (20A + XY)	8
8a. Prophase without heterochromatin blocks dispersed inside the nucleus	9
8b. Prophase with heterochromatic blocks dispersed inside the nucleus	10
9a. Chromocenter formed by a single heterochromatic corpuscle	*Psammolestes tertius*
9b. Chromocenter formed by three heterochromatic corpuscles	*Rhodnius neglectus*
10a. Chromocenter formed by XY sex chromosomes	*Rhodnius nasutus*
10b. Chromocenter formed by XY sex chromosomes and autosomes	11
11a. 45S rDNA probe located in one X sex chromosome	12
11b. 45S rDNA probe located in one autosomal pair	13
12a. Chromocenter formed by XY sex chromosomes and attached bivalent	*Triatoma petrocchiae*
12b. Chromocenter formed by XY sex chromosomes and several bivalents	*Triatoma sordida*
13a. Heterochromatin in 3–4 pairs of autosomes	*Triatoma pseudomaculata*
13b. Heterochromatin in all autosomes	*Triatoma brasiliensis*

**Table 2:** Dichotomous key for species from Rio Grande do Norte, based on cytogenetic data.

Identification Key (CytoKey)	
1. Karyotype with 2n = 21 chromosomes (18A + X_1_X_2_Y)	*Panstrongylus megistus*
2. Karyotype with 2n = 25 chromosomes (22A + X_1_X_2_Y)	*Triatoma rubrofasciata*
3. Karyotype with 2n = 24 chromosomes (20A + X_1_X_2_ X_3_Y)	4
4a. 45S rDNA probe located in one autosomal pair	*Panstrongylus lutzi*
4b. 45S rDNA probe located in one X sex chromosome	*Triatoma melanocephala*
5. Karyotype with 2n = 22 chromosomes (20A + XY)	6
6a. Prophase without heterochromatin blocks dispersed inside the nucleus	*Psammolestes tertius*
6b. Prophase with heterochromatic blocks dispersed inside the nucleus	7
7a. 45S rDNA probe located in one autosomal pair	8
7b. 45S rDNA probe located in one X sex chromosome	9
8a. Heterochromatin in 3–4 pairs of autosomes	*Triatoma pseudomaculata*
8b. Heterochromatin in all autosomes	*Triatoma brasiliensis*
9a. Chromocenter formed by XY sex chromosomes	*Rhodnius nasutus*
9b. Chromocenter formed by XY sex chromosomes more autosomes	*Triatoma petrocchiae*

## Data Availability

All relevant data are within the manuscript.
